# Understanding Plant Social Networking System: Avoiding Deleterious Microbiota but Calling Beneficials

**DOI:** 10.3390/ijms22073319

**Published:** 2021-03-24

**Authors:** Yong-Soon Park, Choong-Min Ryu

**Affiliations:** 1 Biotechnology Research Institute, College of Natural Sciences, Chungbuk National University, Cheongju 28644, Korea; yspark2005@cbnu.ac.kr; 2 Molecular Phytobacteriology Laboratory, Infection Disease Research Center, KRIBB, Daejeon 34141, Korea; 3 Biosystem and Bioengineering Program, University of Science and Technology (UST) KRIBB School, Daejeon 34141, Korea

**Keywords:** beneficial microbiota, communication, multitrophic interaction, plant growth-promoting rhizobacteria, plant social networking system

## Abstract

Plant association with microorganisms elicits dramatic effects on the local phytobiome and often causes systemic and transgenerational modulation on plant immunity against insect pests and microbial pathogens. Previously, we introduced the concept of the plant social networking system (pSNS) to highlight the active involvement of plants in the recruitment of potentially beneficial microbiota upon exposure to insects and pathogens. Microbial association stimulates the physiological responses of plants and induces the development of their immune mechanisms while interacting with multiple enemies. Thus, beneficial microbes serve as important mediators of interactions among multiple members of the multitrophic, microscopic and macroscopic communities. In this review, we classify the steps of pSNS such as elicitation, signaling, secreting root exudates, and plant protection; summarize, with evidence, how plants and beneficial microbes communicate with each other; and also discuss how the molecular mechanisms underlying this communication are induced in plants exposed to natural enemies. Collectively, the pSNS modulates robustness of plant physiology and immunity and promotes survival potential by helping plants to overcome the environmental and biological challenges.

## 1. Introduction

Phytobiome refers to the ecosystem within and surrounding a plant, which comprises diverse organisms including viruses, bacteria, archaea, fungi, oomycetes, other plants and even animals. Since their first appearance in the ecosystem, plants have evolved alongside a variety of beneficial organisms, pathogens and insects. In other words, being sessile organisms, plants have evolved strategies to survive under biotic- and abiotic stresses [1]. The physiology of aboveground plant organs such as leaf and stem differs from that of belowground structures such as the root system, and the difference between two types of plant structures facilitates interaction and communication between biotic stresses [2]. In response to external stimuli such as pathogen and insect attacks, plants have developed a systemic translocational signaling system, in addition to local modulation on immunity. Depending on the types of interactions between biotic communities, the biochemical and physiological fitness of plants has been modified synergistically, antagonistically or neutrally [1].

Interaction with a certain biotic stimulus stimulates the development of defense strategies at a local (infection site) area in plants [3]. Subsequently, defense signals derived from a local region of infected plants are transferred to systemic sites [3]. In addition to the intracellular signals and molecules, the plant-derived signals and molecules act as inter-compartment or inter-plant regulators [4]. To compromise the stress factors, plants orchestrate sophisticated machineries to cooperate with the soil microbiome [5,6,7]. In the rhizosphere, a number of microbes interact with plant roots, which can stimulate plant growth and immunity but also acquire some key nutrients in return [5,8]. Thus, plants and mutualistic microbes communicate with each other, and beneficial interactions between these partners can facilitate defense against invading enemies [9,10].

Multitrophic interactions between plants and microbes (i.e., detrimental insect–plant interactions and beneficial microbe–plant interactions) can be utilized to attenuate plant disease occurrence. For instance, in pepper (*Capsicum annuum*), foliar aphid feeding recruited rhizosphere bacteria and stimulated plant immunity against the leaf spot pathogen, *Xanthomonas axonopodis* [11]. In tobacco (*Nicotiana benthamiana*), whitefly infestation modulated plant immunity to produce endogenous salicylic acid (SA), resulting in the attenuation of *Agrobacterium tumefaciens*-induced gall formation [12]. More recently, activation of plant immunity in tomato (*Solanum lycopersicum*) by four Gram-positive bacteria controlled the incidence of bacterial wilt disease caused by *Ralstonia solanacearum* [13]. In addition, root-associated bacteria triggered the release of plant volatile organic compounds (VOCs), referred to as microbe-induced plant volatiles, and affected the rhizosphere microbiota of neighboring plants [14]. These results indicate that plants interact with beneficial microbes under certain stress conditions to control the response to third-party organisms. In this review, we describe the steps that constitute the plant social networking system (pSNS), show how plants and beneficial microbes communicate with each other, and highlight the strategies and underlying mechanisms of the pSNS. With field applications in mind, we also summarize the technological limitations of pSNS and how these could be surmounted.

## 2. The Plant SNS Hypothesis

**Figure 1:**
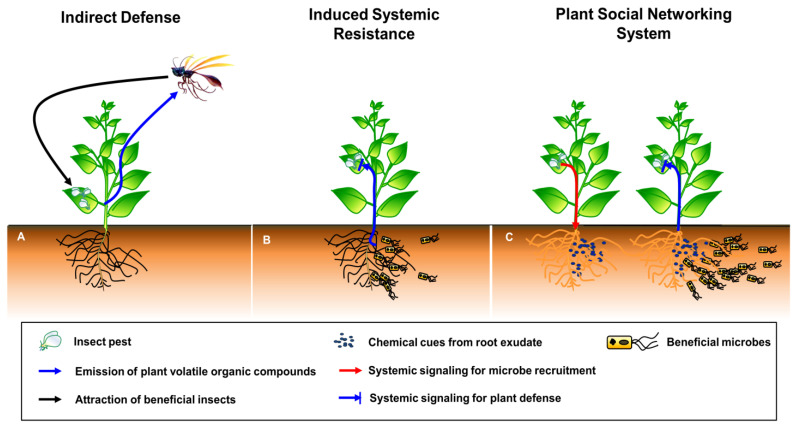
Schematic representation of the plant defense response. (**A**) Indirect defense. Whitefly infestation elicits the plant defense response, which indirectly suppresses the performance of insects attracted by natural volatiles. (**B**) Induced systemic resistance (ISR). Beneficial microbes in the rhizosphere induce systemic signals and systemic-acquired resistance against pathogens and insects. (**C**) Plant social networking system (pSNS). Insect infestation and pathogen infection of aboveground tissues induces the secretion of root exudates into the rhizosphere, which leads to the recruitment of beneficial microbes, thus activating systemic resistance against pathogens and insects.

Recently, the role of plant-associated microbial community has been reviewed in plant–plant communications as wired- and wireless components [15]. In current review, we have focused on the role of plant in modulating SNS against multitrophic stimuli. Therefore, the definition of plant SNS should be clarified first as compared to indirect defense and induced systemic resistance (ISR). Plant defense mechanisms can be classified into two categories: preformed defense and induced defense [16]. Induced defense is normally when they have turned off their defensive strategies to regulate the fitness cost of plants under natural conditions, but plant immunity can be induced by the perception of invading pathogens and insects [16]. Subsequently, during plant–insect interaction, plant defense mechanisms can be similarly divided into two categories: direct defense and indirect defense. In direct defense, plants produce toxic compounds to directly control the population of invading insects. However, in indirect defense, plants attract carnivores that feed on the invading insects, thus indirectly controlling the insect population (Figure 1A [17,18]). The feeding of insects on plant leaves activates the indirect defense of plants, resulting in the activation of elicitors (fatty-acid conjugates, enzymes, cell wall fragments, peptides and esters), plant hormones (SA, jasmonic acid [JA] and ethylene [ET]) and plant volatiles (terpenes, aldehydes, ketones, esters, alcohols and nitrogen compounds) (Figure 1A [19]). These serial events attract natural enemies (predators, parasitic wasps and omnivores), which suppress the insect population.

Induced systemic resistance (ISR) is a form of induced plant defense initiated by beneficial microbes (endophytes, mycorrhizal fungi and plant growth-promoting rhizobacteria) against invading pathogens and insects (Figure 1B). The majority of beneficial microbes are localized in the plant rhizosphere, and several of these microbes promote plant growth and stimulate biotic and abiotic stress resistance [20]. The beneficial microbe-triggered ISR in plants generally suppresses a variety of pathogens and insects [21,22,23].

The induced and indirect defense mechanisms have been thoroughly investigated in plants, both ecologically and biochemically. The most well-established area of research is the role of phytohormones in plant immune signaling. SA, JA and ET function as the main regulators of plant defense responses against pathogens and insects. SA-dependent pathways are activated by biotrophic pathogens and sucking insects, whereas JA- and ET-dependent pathways are induced by necrotrophic pathogens and chewing insects [24,25]. In addition, JA- and ET-related pathways show extensive crosstalk during plant defense responses [24]. In the ISR pathway, while increasing evidence suggests that JA and ET play a pivotal role in suppressing diseases and insects [23,26], more recent studies show that antagonism between SA and JA/ET has been broken down in some cases [27,28].

Here, we propose the plant SNS hypothesis, which is unlike plant indirect defense and ISR (Figure 1C). When plants are attacked by insects or infected by pathogens, certain signals or molecules released by aboveground tissues are transmitted to the rhizosphere, resulting in recruitment of beneficial microbes. These microbes then activate plant defense responses against spatially separated and systemically localized pathogens and insects. The major difference of plant SNS compared with ISR is plant self-modulation against enemy’s attacks through recruiting beneficial microbe-mediated systemic signaling. Indirect defense is not stimulated against plant microbial pathogens, but it can be operated against insect pests. Hereafter, we focus on how plants build the SNS in a step-by-step manner, including the elicitation in a local area, activation and transduction of systemic signals, secretion of bioactive root exudates and chemicals into the rhizosphere, the establishment of a favorable environment (by recruiting beneficial microbes and avoiding plant pathogens), and molecular mechanisms underlying the plant SNS.

## 3. Building Blocks and Molecular Mechanisms of the Plant SNS

**Figure 2:**
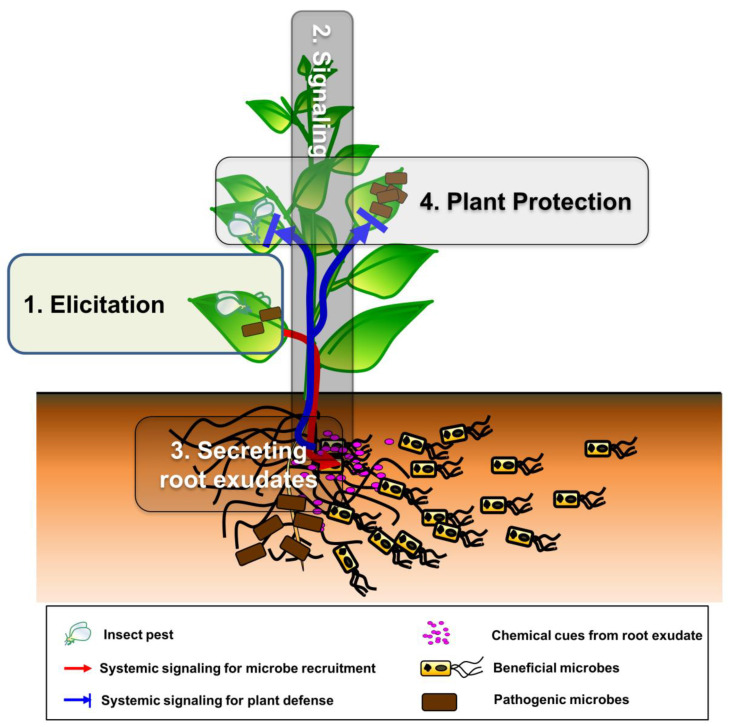
Schematic representation of the plant SNS. The events at each step of multitrophic interactions include (1) elicitation; (2) systemic signaling; (3) root exudate secretion; and (4) plant protection.

A stepwise procedure for building the plant SNS (Figure 2) is described below. Here, we describe the steps that constitute the plant social networking system (pSNS); summarize, with evidence, how plants and beneficial microbes communicate with each other in each step; and finally, highlight the strategies and underlying mechanisms of the pSNS.

### 3.1. Elicitation: Plant Incuction in a Local Area by Insect and Pathogen Attacks

Plants are constantly exposed to diverse pathogens and insects, and unlike animals, plants have developed effective immune strategies to defend themselves against these biotic stressors by recognizing common and species-specific determinants on pathogens and insects. Interestingly, plants have developed an innate immune response that resembles the animal immune system. Plants can detect common foreign signals such as pathogen/microbe/herbivore-associated molecular patterns (PAMPs/MAMPs/HAMPs) via pattern-recognizing receptors (PRRs) [29,30]. The well-characterized plant PAMPs include flg22 (the N-terminus of bacterial flagellin), elf18/elf26 (the N-terminus of bacterial elongation factor Tu), peptidoglycans (a component of bacterial cell walls), and chitin (a component of fungal cell walls) [29,30,31,32,33]. In addition, plants can also perceive herbivore-derived precursors to form fatty acid–amino acid conjugates as HAMPs including volicitin, inceptins, caeliferin and bruchin [34,35,36,37].

The perception of MAMPs and HAMPs by plant cells induces an influx of extracellular calcium ions (Ca^2+^), production of reactive oxygen species (ROS) [38,39] and activation of mitogen-activated protein kinase (MAPK) cascades that play an important role in the regulation of downstream signaling [40,41,42]. Phytohormone signaling pathways represent a critical component of plant defense responses against pathogens and insects, as discussed above. Among various plant hormones, SA, JA and ET are the key regulators of plant defense [24,25]. These plant hormones are also utilized as signaling molecules to activate plant systemic immune responses.

### 3.2. Signaling: Activation and Transduction of Systemic Signaling Molecules

After recognizing a microbial pathogen or an insect pest’s determinants, plants generate signal molecules that translocate from the attack site (local site) to systemic organs. Here we summarize the signaling molecules that play an important role in this system. The signaling molecules include SA, MeSA, JA, JA derivatives, volatile organic compounds, and lipid-derived molecules.

#### 3.2.1. SA and Methyl Salicylate

SA and its volatile derivative, methyl salicylate (MeSA), are strong systemic signal candidates. The first reports strongly support that SA is normally required for activating systemic resistance in tobacco and cucumber (*Cucumis sativus*) [43,44,45]. Because the first several reports supported the potential role of SA as a systemic signal, subsequent studies revealed its role in systemic signal transduction using mutant lines [46,47,48,49,50,51]. Further investigation showed that glycosylated SA compounds, such as SA-glycosyd (SAG), and MeSA are accumulated by plant cells upon pathogen infection [52,53,54]. An early study showed that MeSA is transmitted from *Tobacco mosaic virus* (TMV)-infected donor (emitter) plants to healthy (receiver) plants, resulting in a 30% decrease in TMV lesion size [55]. Several grafting experiments revealed that SA is converted into MeSA in infected leaves, which is subsequently translocated to distal systemic leaves, where it is converted back to SA to activate systemic resistance against biotrophic pathogens and sucking insects [56,57,58,59,60,61]. SA-dependent systemic signaling is mainly activated by sucking (phloem-feeding) aphids and spider mites [62].

#### 3.2.2. JA and Its Derivatives

Unlike SA, JA and its derivatives, methyl jasmonate (MeJA) and jasmonoyl isoleucine (JA-Ile), function as signaling molecules in plant defense against necrotrophic pathogens and chewing insects [24,63,64]. JA is synthesized in vascular bundles, which also host prosystemin biosynthesis, and JA, systemin and prosystemin are related to each other in a double feedback manner [65,66]. Upon the attack of insect herbivores, polyunsaturated fatty acids (PUFAs) are released from the plant membrane lipids by lipases such as defective in anther dehiscence 1 (DAD1) and dongle (DGL), while PUFAs such as linolenic acid (C18:3) and linoleic acid (C18:2) are oxygenated by lipoxygenases (LOXs), which catalyze the synthesis of a large variety of oxylipins, including JA and green leafy volatiles (GLVs), through 6–7 independent pathways [67,68,69,70]. In addition to JA, conjugates of JA also act as systemic signals [71]. For example, JA-Ile acts as an active signal for the regulation of defense responses rather than JA itself [72,73], and MeJA is critically involved in diverse interactions as a key signal [74].

#### 3.2.3. Gaseous Signals VOCs

Besides the volatile forms of SA and JA, other VOCs are also released by plants in response to attack by a variety of insect herbivores [75,76,77,78]. More than 1000 VOCs have been identified in plant tissues including flowers, and vegetative organs [79,80]. Plant-derived VOCs are the main signals involved in multitrophic interactions. Among these VOCs, the main volatiles (C6-alcohols and C6-aldehydes) are derived from GLVs by the action of hydroperoxide lyase (HPL) through the octadecanoic-derived LOX pathway, and some of the GLVs are required for direct defense, which explains why the deletion of the HPL gene in transgenic potato (*Solanum tuberosum*) decreased the resistance to aphids [81]. Cis-3-hexen-1-ol is emitted upon herbivore attack to attract a generalist predator under field conditions [76]. Trans-2-hexenal is also released upon herbivore-induced wounding, and its emission influences the accumulation of sesquiterpenes in wounded *Arabidopsis thaliana* and cotton (*Gossypium hirsutum*) plants, suggesting that GLVs are involved in intra- and inter-plant defense signaling pathways in response to diverse insects [82,83]. VOCs emitted upon insect infestation are referred to as herbivore-induced plant volatiles (HIPVs). Other groups of VOCs, including monoterpenes (limonene, linalool and ocimene), sesquiterpenes (bergamotene, carphyllene and farnesene), MeSA and MeJA, are released from the wound site within 24 h of herbivore attack [79,80,84,85].

#### 3.2.4. Lipid-Derived Signals

Unlike the plant VOCs induced by herbivore attack, plants generate lipid-based signal molecules that translocate from one tissue to another. The defective in induced resistance 1 (DIR1) gene encodes a 7 kDa lipid transfer protein, which is localized in petiole exudates of pathogen-infected leaves either as an oligomer or in a complex with other proteins [86,87]. DIR1 interacts with other lipid-derived signaling compounds such as azelaic acid (AzA), dehydroabietic acid (DA), glycerol-3-phosphate (G3P) and MeSA [86,88,89,90].

G3P functions as an important signaling molecule in systemic resistance, and localizes to the cytosol and chloroplast. Several mutant-based studies have proven the function of G3P as a systemic signal. In *Arabidopsis*, disruption of the suppressor of fatty acid deficiency 1 (SFD1) gene, which encodes dihydroxy acetone phosphate (DHAP), elucidated the role of G3P as a systemic signal [91]. The sfd1 mutant showed low levels of SA accumulation and pathogenesis-related 1 (PR1) expression in distal tissues [91,92]. In other studies, mutation of SFD2, fatty acid desaturase 7 (FAD7), monogalactosyl synthase 1 (MGD1) and glycerol insensitive 1/nonhost 1 (GLI1/NHO1) abolished systemic responses [88,93].

AzA, a nine-carbon dicarboxylic acid, is the final product of lipid peroxidation under biotic stress [94]. The notion that AzA functions as a signaling molecule in systemic resistance was first verified by Jung and colleagues [89]; the authors showed that AzA acts as a priming molecule, and SA levels are elevated in distal leaves after infection by *Pseudomonas syringae* pv. maculicola strain *Pma*DG3 or upon the application of exogenous AzA, loss of systemic resistance of AZI1 induced by AzA is observed by gene disruption analysis [89]. In contrast to the results of Jung and colleagues [89], other studies could not elucidate the priming effect of AzA on SA accumulation in systemic leaves [95]. The possible routes of AzA biosynthesis in plants under biotic stress conditions are complex and controversial, indicating that the bacteria-derived nine-carbon product (i.e., AzA) of plants is potentially synthesized by non-enzymatic pathways, although LOXs are mainly required for the accumulation of lipid derivatives [94]. Recent studies show that galactolipids produce AzA via the ROS-mediated pathway [94,96]. The aboveground elicitation by pathogen and insect attacks leads to the activation of plant signaling transduction pathways, resulting in the secretion of root exudates that modulate rhizosphere microbiota and plant immunity.

### 3.3. Secreting Root Exudates: Plant Secretion of Bioactive Root Exudates and Chemicals into the Rhizosphere

The chemical signals of plants infected with pathogens and insects can be translocated to the root and affect compositions and contents of root exudates in the rhizosphere. The concept of plant–microbe interactions in the rhizosphere is not novel; however, the exact roles and composition of plant-derived root exudates remain unclear. Rhizodeposits released into the rhizosphere by plant roots include root exudates, mucilage, lysates released from wound sites and senescing cells that contain mono-, polysaccharides, organic acids, phenolic compounds, amino acids, and proteins [97,98]. Root exudates generated from the meristematic zone of root tips are the major components of rhizodeposits, and contain sugars, amino acids, organic acids, phenolics, alcohols, polypeptides and proteins [97,99]. Chemical compounds in root exudates play a pivotal role in acquiring mineral nutrients, attracting beneficial microbes and suppressing deleterious pathogens in the rhizosphere [5,100,101,102,103]. Here, we highlight the diverse compounds in root exudates released in response to nutrient limitations, pathogen infection and insect infestation, thus attracting microbes and other biotic stimuli.

#### 3.3.1. Secretion of Strigolactones (SLs), Flavonoids and Coumarins under Nutrient Limitation Conditions

Under nutrient-deficient conditions, plants secrete large amounts of SLs, flavonoids and coumarins into the rhizosphere. The recently discovered plant hormone, SL, is synthesized from a carotenoid precursor and is secreted by plants under phosphate-deficient conditions to recruit arbuscular mycorrhizal (AM) fungi, resulting in phosphate uptake [104,105]. SLs affect the interactions between the host plant and AM fungi; abolishing SL biosynthesis decreases the colonization efficiency of *Gigaspora rosea* [106]. Flavonoids are a group of secondary metabolites secreted into the rhizosphere under phosphate- and nitrogen-limiting conditions. Flavonoids participate in the interaction between legumes and *Rhizobium* spp. and between actinorhizal plants and *Frankia* spp.; plants utilize nitrogen fixed by the bacteria, and in turn bacteria obtain carbon sources from the plant [107]. Coumarins are low-molecular-weight (LMW) secondary metabolites similar to flavonoids and are involved in plant–pathogen interactions as antimicrobial compounds [108]. However, recent studies revealed the function of coumarins as components of root exudates released by plants under nutrient-deficient conditions. *Arabidopsis* roots secrete diverse coumarins, including scopoletin, esculetin, fraxetin and sideretin, under iron limitation conditions. Additionally, genetic analyses revealed that *Arabidopsis* mutant lines grown in iron-deficient soils lack the ability to secrete or synthesize coumarins [109,110,111,112]. Roots of the annual grass *Avena barbata* secrete tryptophan and sucrose into the rhizosphere, and the tryptophan residue located close to the lateral roots potentially interacts with indole-3-acetic acid (IAA) to modulate lateral root initiation [113].

#### 3.3.2. Secretion of Malic Acid and Phenolic/Organic Acid Compounds upon Pathogen Infection

The infection of *Arabidopsis* leaves by *P. syringae* pv. tomato (*Pto*) DC3000 facilitates the secretion of malic acid into the rhizosphere, which attracts *Bacillus subtilis* FB17 [5]. In addition, *Pto* infection increases the secretion of long-chain organic acids (pentadecanoic acid, hexadecanoic acid, palmitoleic acid, octadecanoic acid and arachidic acid) and amino acids (isoleucine, leucine, methionine, proline, tryptophan and ornithine) [114]. Infection by soil-borne pathogens modifies the root exudates of host plants and alters the microbial composition of the rhizosphere [20,115]. Infection of soybean (*Glycine max*) roots by *Pythium ultimum* increased the concentrations of phenolic and organic acids such as vanillic acid, p-coumaric acid and fumaric acid by 4-fold [116]. Additionally, antimicrobial compounds such as caffeic acid ester and rosmarinic acid are released by sweet basil (*Ocimum basilicum*) in response to infection by *Pythium* spp. [117]. *Fusarium* spp. induce the secretion of antifungal phenolics from barley (*Hordeum vulgare*) roots [118]. Infection of tobacco roots by the root-knot nematode *Meloidogyne incognita* leads to the accumulation of the defense-related compound nicotine in aboveground tissues, which is effectively utilized to attenuate foliar herbivores [119]. In potato, the powdery scab pathogen *Spongospora subterranea* facilitates the secretion of root exudates containing 24 different kinds of LMW compounds such as amino acids, sugars and organic acids, among others [120]. In tobacco, infection by bacterial wilt and black shank pathogens increases the secretion of amines, alcohols, lipids, sugars and esters in root exudates, and these compounds modulate the pathogen-antagonizing microbes [121].

#### 3.3.3. Secretion of Benzoxazinoids and SA upon Insect Infestation

Insect infestation of aboveground and belowground plant tissues stimulates the secretion of root exudates into the rhizosphere to recruit microbes or suppress disease. Maize (*Zea mays*) roots secrete benzoxazinoids (BXs) into the rhizosphere [122,123]. However, the role of BXs in the context of exudates and their effect on soil microbial composition has not yet been investigated. Infestation of maize leaves by *Spodoptera frugiperda* induces the secretion of BXs into the rhizosphere, which affects the soil microbiota [124]. Insect infestation also leads to the production of certain volatiles in root exudates. Western corn rootworm feeding induces the accumulation of (E)-β-caryophyllene in belowground tissues, which attracts entomopathogenic nematodes [125]. In *Citrus* spp., four terpene volatiles were detected in root exudates after infestation by root weevil (*Diaprepes abbreviatus*) [126].

Whitefly infestation of aboveground tissues elicits the SA-dependent pathway in belowground tissues to suppress the soil-borne pathogen *R. solanacearum* in pepper [127] and *A. tumefaciens*-induced crown gall formation in tobacco [12]. Similarly, the attack of aboveground tissues by aphids modulates defense responses in belowground tissues via SA- and JA-dependent pathways to control the population of foliar bacterial pathogens and soil-borne pathogens [11]. Moreover, in potato, aphid (*Myzus persicae*) infestation of aboveground tissues induces the secretion of root exudates into the rhizosphere, showing that root exudates from aphid feeding on aboveground into the belowground and Neprilysin-1 of cyst nematode (*Globodera pallida*) are highly linked [128]. Moth-induced defoliation of the aboveground plant parts of the subarctic mountain birch (*Betula pubescens*) results in the release of carbon-rich compounds into the rhizosphere to modulate ectomycorrhizal fungi [129].

### 3.4. Plant Protection

Since root exudates containing diverse compounds and molecules are secreted into the rhizosphere, they can potentially generate signals that increase plant protection. In this part, we introduce three scenarios: recruitment of beneficial microbes by root exudates, antibiosis and antimicrobial compounds, and induced systemic resistance (ISR).

#### 3.4.1. Recruitment of Beneficial Microbes by Root Exudates

One of the main functions of plant root exudates is the recruitment of beneficial microbes, which will protect plants under biotic and abiotic stress conditions. The composition of root exudates generally varies with soil nutrient status, disease incidence and abiotic stresses [130], and affects the soil microbial composition [99,131]. Studies show that biotic stresses can cause the secretion of chemicals into root exudates, thus attracting other microbes. For example, *Fusarium oxysporum*-infected tomato plants recruit *Proteobacteria*, *Actinobacteria* and *Firmicutes* [132], and *Botrytis cinerea* infection induces the accumulation of *Trichoderma harzianum* in the rhizosphere of tomato and cucumber plants [133]. In *Arabidopsis*, infection of leaves by the downy mildew pathogen *Hyaloperonospora arabidopsidis* leads to the recruitment of beneficial microbes such as genus of *Xanthomonas*, *Stenotrophomonas*, and *Microbacterium* [5,134,135,136], while infection by *Pto* DC3000 facilitates the attraction of *B. subtilis* FB17 [5,136] and leads to the assembly of a beneficial rhizosphere microbiome [114]. In sugar beet (*Beta vulgaris*), infection by *Rhizoctonia solani* alters the microbiome composition and attracts bacteria belonging to the families *Oxalobacteraceae*, *Burkholderiaceae*, *Sphingobacteriaceae* and *Sphigomonadaceae* [137].

In addition to pathogen infection, insect infestation has also been shown to attract beneficial microbes. In pepper, aboveground aphid feeding recruits beneficial microbes in the rhizosphere; thus, the population density of *Bacillus subtilis* GB03 was significantly higher in aphid feeding plants than in control plants, but the population density of *Pseudomonas protegens* Pf-5 was not affected by aphid feeding [11]. Similarly, whitefly infestation of pepper plants stimulates the recruitment of Gram-positive bacteria and fungi in the rhizosphere [127]. In maize, chewing by *S. frugiperda* alters the soil microbiota [124]. In *Brassica napus*, infestation of belowground tissues by cabbage root fly (*Delia radicum*) attracts four bacterial genera (*Bacillus*, *Paenibacillus*, *Psedomonas* and *Stenotrophomonas*) in the rhizosphere [138]. Root herbivory of bentgrass (*Agrostis* spp.) and clover (*Trifolium* spp.) plants by *Tipula paludosa* can utilize *Pseudomonas* compared with non-infested plants [139].

#### 3.4.2. Antibiosis and Antimicrobial Compounds

Beneficial microbes recruited by the secretion of plant root exudates produce antibiotics, which can be utilized as biological control agents against harmful pathogens via a phenomenon known as antibiosis [140]. The major antibiotics produced by bacteria include hydrogen cyanide (HCN) [141], phenazine-1-carboxylic acid [142], phenazine-1-carboxyamide [142], 2,4-diacetyl phloroglucinol (Phl) [143], pyoluteorin [144] and pyrrolnitrin [145]. Phenazine-1-carboxylic acid produced by *Pseudomonas fluorescens* 2–79 in the wheat rhizosphere attenuates the disease-causing fungus *Gaeumannomyces graminis* var. tritici, and mutant analysis of phenazine-1-carboxylic acid showed that its mutation partially contributes to the alleviation of disease symptoms [146]. HCN, a volatile antibiotic produced by *P. fluorescens* CHA0, negatively regulates the fungal pathogen of black root rot, *Thielaviopsis basicola*, in tobacco [147].

In addition, lipopeptides such as fengycin, surfactin and iturin are LMW compounds produced by *B. subtilis* strains. These lipopeptides can directly suppress pathogenic fungi under pre- and post-harvest conditions [148]. Iturin A derived from *B. subtilis* strains PCL1608 and PCL1612 directly controls two fungal pathogens, *Fusarium oxysporum* and *Rosellinia necatrix* [149]. Surfactin produced by *B. subtilis* strain 6051 acts as a biocontrol agent in response to pathogenic bacteria [150].

Moreover, some studies have shown that root exudates secreted by plants exposed to pathogens exhibit direct antimicrobial activity. Two types of LMW antimicrobial compounds, phytoanticipins and phytoalexins, are involved in the direct suppression of pathogens [151,152]. The distinction between these two types of compounds is, however, difficult because pathogen infections sometimes induce the accumulation of phytoalexins as well as phytoanticipins [151]. Root exudates of barley plants infected by *Fusarium graminearum* contain aromatic compounds including five phenylpropanoids, such as t-cinnamic acid, p-coumaric acid, ferulic acid, syringic acid and vanillic acid, which exhibit direct antifungal activity and inhibit the germination of *F. graminearum* macroconidia [153]. In addition, scopoletin, a coumarin from plants, directly inhibits *F. oxysporum* and *Verticillium dahliae* [154]. Root exudates of pine tree (*Pinus resinosa*) associated with the ectomycorrhizal fungus *Paxillus involutus* contain ethanol-soluble compounds that act as antifungal molecules, suppressing the sporulation of *F. oxysporum* by 80% [155].

#### 3.4.3. Induced Systemic Resistance

Beneficial microbes, including bacteria and fungi, can activate ISR, and its initiation signals translocate the whole plant to suppress the invading pathogens/insects (Figure 1B). *Pseudomonas* spp., *Bacillus* spp., *Trichoderma* spp. and mycorrhizae have been shown to enhance plant immunity [3,156,157,158]. Here, we describe how beneficial microbes induce the plant defense response against pathogens and insects in aboveground tissues. This phenomenon was first proven by some research groups in 1991: in carnation (*Dianthus caryophyllus*) plants, colonization by *P. fluorescens* WCS417r elicited resistance, which led to the accumulation of antimicrobial phytoalexins in response to the aboveground infection by the fungal pathogen *F. oxysporum* [159]; in cucumber plants, root colonization of *Pseudomonas putida* and *Serratia marcescens* 90–166 suppressed the symptoms of anthracnose caused by the fungal pathogen *Colletotrichum orbiculare* [160,161]. These two strains also stimulate ISR against several pathogens including *F. oxysporum* (*Fusarium* wilt pathogen; [162]), *P. syringae* (bacterial angular leaf spot pathogen; [163]), *Cucumber mosaic cucumovirus* (CMV) [164] and *Erwinia tracheiphila* (cucurbit wilt pathogen; [165]). The activation of ISR by rhizobacteria suppresses the disease caused by *P. syringae* pv. tabaci [166]. Some bacteria activate ISR in tobacco to suppress blue mold disease caused by *Peronospora tabacina* [167].

In addition to activating ISR against pathogens, mycorrhizal fungi also exert negative effects of herbivore performance [168,169]. In tomato, *B. subtilis* BEB-DN promotes the activation of ISR in aboveground tissues against *Bemisia tabaci* infestation [170], and pre-inoculation of tomato plants with *B. subtilis* triggers resistance against *Bemisia tabaci* under greenhouse conditions [171]. A field study showed that the population size of cucumber beetles is significantly decreased by several bacteria such as *P. putida* strain 89B-61, *S. marcescens* strain 90–166, *Flavimonas oryzihabitans* strain INR-5 and *Bacillus pumilus* strain INR-7 [172]. However, insect performance is positively regulated by *P. fluorescens* WCS417r in *Arabidopsis* [173]. The molecular mechanism of ISR has been well established in *Arabidopsis*, for example, using *P. fluorescens* WCS417r; systemic resistance triggered by *P. fluorescens* WCS417r activates the deposition of callose and up-regulates the expression of JA/ET-related genes, *PDF1.2* and *VSP2* [174,175,176]. These data suggest that JA- and ET-mediated pathways promote ISR, especially in *Arabidopsis* and tomato [177,178]. On the other hand, some studies rely on the activation of SA-dependent pathways triggered by certain rhizobacteria [179,180]. The summary of plant SNS steps is listed in Table 1.

### 3.5. Molecular Mechanisms Underlying the Plant SNS

In this part, the molecular mechanisms of plant SNS have been introduced in several recent studies. In pepper, whitefly infestation of aboveground plant parts elicits SA and JA signaling both in above- and below-ground tissues, and alters the microbiome assembly, leading to the attenuation of *Xanthomonas axonopodis* pv. vesicatoria (*Xav*) and *R. solanacearum* SL1931 [127]. Interestingly, although insect feeding does not cause any physiological changes in aboveground tissues, the root biomass is augmented, indicating that certain molecules and/or signals in aboveground organs are potentially transferred to belowground tissues [127]. Increase in root biomass has been consistently observed in several studies [12,181,182].

Whitefly infestation stimulates SA-mediated signaling attracting beneficial microbes, which would reduce the incidence of *A. tumefaciens*-induced gall formation in both above- and belowground tissues. Song and colleagues monitored the level of endogenous SA in whitefly-infested tobacco plants, and genetic analysis supported the phytohormone assays showing that the SA biosynthetic gene, *ICS1*, plays an important role in the accumulation of SA [12]. SA directly suppresses the expression of pathogenicity and virulence-related genes in *Agrobacterium*. Moreover, the content of IAA in whitefly-infested tobacco plants increased upon *Agrobacterium* inoculation [12], suggesting that IAA plays a pivotal role in the positive regulation of root biomass. This result was supported by the induction of auxin response genes and nutrient transporter genes in whitely infested roots [181].

Similar to whitefly infestation, aboveground aphid feeding elicits SA and JA signaling pathways, recruits *B. subtilis* GB03 and prevents the soil-borne bacterial pathogen *R. solanacearum* SL1931, thus priming pepper immunity in response to the pathogenic bacterium *Xav* and a non-pathogenic bacterium *X. axonopodis* pv. glycines (*Xag*). In a recent study, the severity level of disease caused by *Xav* was reduced by approximately 4-fold in aphid feeding plants compared with control plants, and the hypersensitive response index against *Xag* was significantly delayed in aphid feeding plants [11].

In addition to insect-induced plant SNS, pathogen-triggered plant SNS has been recently investigated. *B. cinerea*, a foliar fungal pathogen, secretes peroxidases and oxylipins as chemoattractant molecules into the roots of tomato plants for attracting *Trichoderma harzianum* T22 and inhibiting *F. oxysporum* [133]. Inoculation of *Arabidopsis* leaves with the pathogenic bacterium *Pto* DC3000 stimulates intra-plant long-distance signaling, activates the malate transporter AtALMT1 and leads to the secretion of malic acid. Malic acid in root exudates attracts *B. subtilis* FB17, which colonizes plant roots. These sequential signaling events enhance ISR to modulate defense responses against *Pto* DC3000 [136].

In sugar beet, inoculation of roots with the fungal pathogen *R. solani* induces the plants to respond to oxalic acid. Ribosomal RNA-based analyses revealed that *R. solani* infection significantly increased the population densities of *Oxalobacteraceae*, *Burkholderiaceae*, *Sphingobacteriaceae* and *Sphingomonadaceae* in the rhizosphere, and up-regulated the expression of genes involved in the regulation of bacterial stress responses, resulting in protection against *R. solani* [137].

## 4. Technological Limitations, Fundamental Issues, and Potential Troubleshooting Approaches

Although the proposed plant SNS represents a good strategy for stimulating plant growth and immunity, certain technical and experimental limitations and unanswered questions need to be addressed. Firstly, building multitrophic interactions is quite complicated and therefore must be adapted for very narrow studies. To investigate the interactions among host plants, microbes and pathogens/insects, a comprehensive analysis strategy is needed. This requires multidisciplinary omics-based tools in diverse fields, such as plant biochemistry, plant genetics, microbiology, genomics, transcriptomics, metabolomics, metagenomics and bioinformatics, for the elucidation of specific compounds in root exudates of pathogen-infected plants [114,124,183].

Secondly, to isolate and characterize composition of root exudates, artificially designed experimental procedures based on well-established systems are needed under laboratory and greenhouse conditions. For example, when whiteflies feed on plant leaves, the collection of root exudates from the rhizosphere soil is not easy. To fill the gap in the current situation, an in vitro bioassay has been alternatively invented [12,136]. Additionally, root exudate profiles under each condition need to be evaluated for connecting the plant phenotypes. Furthermore, after the isolation and characterization of compounds and molecules in root exudates, the functions of these compounds must be validated under natural conditions.

Thirdly, a single strain of a pathogen is sometimes not sufficient for manipulating the potential pathogens and insects. Accumulating evidence suggests that a certain bacterial strain, which has no effect on controlling plant defense responses, is effective when inoculated with other bacteria [5,184]. This is supported by the finding that inoculation of multiple strains is more effective in enhancing pathogen resistance than inoculation of a single strain [5,185,186]. Moreover, a recent study showed that microbial synthetic communities produce ISR-promoting substances that can be used as inoculants [186]. Diverse synthetic communities effectively suppress Fusarium wilt disease in tomato [186]. To extend the usage of synthetic community approaches, a variety of combinations of microbes have been evaluated for resistance to pathogens and insects. The elucidation of keystone taxa in complex synthetic microbial communities is very important for effective control of pathogens and insects.

Finally, the proposed plant SNS is essential for conclusively understanding the beneficial microbe-driven systemic resistance in plants exposed to pathogens and insects, as well as for the isolation and characterization of specific genes, traits, compounds/chemicals and microbial strains; however, whether studies on the plant SNS will generate reproducible results under field conditions remains unclear. For example, a single bacterial strain as well as synthetic communities of bacteria elicits negative effects on pathogen in vitro; however, the results may be different under field conditions [187]. Therefore, the reproducibility of plant SNS is a major issue for manipulating plant systemic defense responses against pathogens and insects. This suggests that plant SNS-derived ISR should be sustainably investigated and monitored in a variety of multitrophic interactions to improve agriculture in the future.

## 5. Future Perspectives

Plants serve as the major food source for humans, and an increase in plant yield is needed in the near future to meet the needs of the growing world population [188]. Plants are constantly exposed to diverse biotic and abiotic stresses, and therefore have developed sophisticated strategies for overcoming unfavorable conditions. Although many approaches have been used to maintain and produce more edible plants, increasing the plant yield remains a challenge because of natural disasters and invasion by insects and pathogens [189]. Approximately 10–16% of crop plants have been devastated by pathogens and insects, and the usage of chemicals has also increased substantially in the last few decades [190]. One of the proposed ecofriendly methods for increasing plant yield, despite the negative impact of pathogens and insects, is the plant SNS. To decrease the amount of chemical agents needed to control pathogens and insects, it is important to isolate and characterize certain genes, evaluate plant defense traits and beneficial microbe-related traits, and investigate the microbial community in a variety of plant species. If the plant SNS shows reproducible results in the field, this strategy will enhance our understanding of the ecological, economical and industrial aspects of agriculture.

## Figures and Tables

**Table 1:** Summary of plant SNS steps described in this review.

Steps of Plant SNS	Triggers/Determinants	Effect/Mechanisms on Plant	References
1. Elicitation	PAMPs/MAMPs/HAMPs: flg22, elf18/elf26, peptidoglycans, chitin, volicitin, inceptins, caeliferin, and bruchin	Plant pattern receptors perceive PAMPs/MAMPs/HAMPs	[29,30,31,32,33,34,35,36,37]
	Ca^2+^, ROS, MAP Kinase cascades, and phytohormones	Regulation of plant defense responses	[38,39,40,41,42]
2. Signaling	SA and Me-SA	Activating systemic resistance against biotrophic pathogens and sucking insects	[43,44,45,56,57,58,59,60,61,62]
	JA, MeJA, and JA-Ile	Defensive signal against necrotrophic pathogens and chewing insects	[63,71,72,73,74]
	Volatile organic compounds (VOCs): C6-alcohol, C6-aldehydes, cis-3-hexen-1-ol trans-2-hexenal, monoterpenes (limonene, linalool, ocimene), and sesquiterpenes (bergamotene, carphyllene and farnesene)	Released by plants in response to a variety of insects	[75,76,77,78,81,82,83,84,85]
	Lipid-derived signals: DIR1, G3P, and AzA	Signaling molecules to activate systemic defense responses to pathogens	[86,87,89,91,92,93,94,95]
3. Secreting root exudates	Stringolactones, flavonoids, and coumains	Secretion under phosphate- and nitrogen-deficient conditions. Effect on the interaction between plant and AM fungi	[104,105,106,107,108,109,110,111,112]
	Malic acid, phenolic compounds, and organic acids	Secretion after infection with bacterial and fungal pathogens and nematodes	[5,114,116,117,118,119,120,121]
	Benzoxazinoids and SA	Secretion upon insect infestation	[11,12,122,123,124,125,126,127,128,129]
4. Plant protection	Beneficial microbes by root exudates	Recruitment of beneficial microbes from plants infected with pathogens and insects	[5,11,114,124,127,132,133,134,135,136,137,138,139]
	Antibiosis and antimicrobial compounds: hydrogen cyanide, phenazine-1-carboxylic acid, phenazine-1-carboxyamide, 2,4-diacetyl phloroglucinol, pyoluteorin, pyrrolnitrin, phenazine-1-carboxylic acid, t-cinnamic acid, p-coumaric acid, ferulic acid, syringic acid, vanillic acid, scopoletin, and ethanol-soluble compounds	Direct suppression of pathogens and insects by antibiotics, lipopeptides, phenylpropanoids and ethanol-soluble compounds	[140,141,142,143,144,145,146,147,148,149,150,151,152,153,154,155]
	Microbes elicit induced systemic resistance	Activation of broad spectrum plant immunity against pathogens and insects	[159,160,161,162,163,164,165,166,167,168,169,170,171,172,173,174,175,176,177,178,179,180]
